# Delphi method validation of a tool for planning home visits in palliative care: palliative care parametrisation tool

**DOI:** 10.3332/ecancer.2026.2056

**Published:** 2026-01-16

**Authors:** Ariel Cherro, Adriana Fernandez, Horacio Gonzalez, María Victoria Fasano, Lucas Morando

**Affiliations:** 1 Care Home, División Cuidados Paliativos de Nutri Home S.A., CP 1428 - Ciudad Autónoma de Buenos Aires, Argentina; 2 Consejo de Cuidados Paliativos, Sociedad Argentina de Medicina, C1181ACK - Ciudad Autónoma de Buenos Aires, Argentina; 3 Universidad Nacional de Mar del Plata, Cátedra de Medicina Interna y Campos Clínicos II, Mar del Plata, Provincia de Buenos Aires, Argentina; 4 Universidad Nacional de La Plata, Cátedra de Nutrición Humana, B1900 - La Plata, Provincia de Buenos Aires, Argentina; 5 Instituto de Desarrollo e Investigaciones Pediátricas (IDIP), Hospital de Niños La Plata, CP 1900 - La Plata, Provincia de Buenos Aires, Argentina

**Keywords:** palliative care, Delphi method, home visits, nursing score, parametrisation tool, validation study

## Abstract

**Background:**

Palliative care parametrisation tool (PCPT) was developed to standardise the planning of home visits in palliative care, specifically for the adult oncology population in Argentina. This study aimed to validate the nursing score (NS) component of the tool using the Delphi method.

**Methods:**

The NS was designed to quantify the caregiving burden based on patient-related factors. Two Delphi rounds were conducted with 19 expert palliative care professionals from Argentina, including 13 physicians and 6 nurses. The content validity of the NS was assessed using the Aiken V test and consensus was reached on the score assigned to each item.

**Results:**

The Aiken V test indicated high levels of concordance between experts for all categories evaluated, with statistical significance (*p* < 0.001). Categories such as ‘Simple Wound Care,’ ‘Complex Wound Care’ and ‘Socio-family Issues’ achieved a perfect Aiken V index (1.00). The Delphi rounds further refined the NS, achieving over 84% agreement for all categories, with two items reaching 100% consensus.

**Conclusion:**

The NS is a valid instrument for determining the number of visits by nurses in home palliative care. The use of parameterisation tools like PCPT aligns with literature recommendations and offers a robust framework for optimising care provision. Future studies are needed to validate the remaining components of the tool.

## Introduction and objective

Palliative care is defined as specialised medical care aimed at improving the quality of life of patients with serious illness. This specialty focuses on relieving symptoms and stress associated with life-limiting diseases, regardless of the stage of the illness or the need for other therapies. Palliative care is holistic, addressing the physical, emotional and social dimensions of the care process [1].

The use of home-based palliative care (HPC) provided by interdisciplinary teams is increasing [2–4], supported by growing evidence of its positive impact on improving quality of life and reducing costs [5, 6].

In the literature, no standardised method has been reported for determining the number of visits to be provided by each member of an interdisciplinary team to an individual patient. The literature on this topic reports that home nursing visits are often determined based on intuition, clinical guidelines, previous experiences with similar cases, healthcare coverage, coordination support and peer recommendations, among other factors [7–10]. This approach can be improved. Some authors have reported enhancements in the decision-making process through standardised visit planning frameworks that integrate dynamic visit scheduling and optimised nursing route planning [11].

The literature suggests that the frequency of palliative home nursing visits should be based on specific patient factors and needs. For example, lower scores on the Palliative Performance Scale, the presence of pain or discharge from a hospital setting have been associated with more frequent visits [12]. These innovative approaches enable a more efficient allocation of resources while maintaining a high standard of care [12].

The present study was carried out by an interdisciplinary HPC team with 15 years of experience, currently providing services in 41 cities across Argentina. On average, 1,600 patients are treated annually, with 70% of whom have oncological conditions and 96% are adults (over 18 years old).

To standardise the number of visits required from each professional in the interdisciplinary palliative care team for a given patient, a palliative care parametrisation tool (PCPT) was developed. This novel instrument integrates validated and preexisting scales—such as the Edmonton Symptom Assessment System (ESAS) and Eastern Cooperative Oncology Group (ECOG)—as well as a newly created, ad hoc nursing score (NS). The PCPT was designed to help care teams determine the appropriate number of visits by physicians, nurses, psychologists and physiotherapists. These four professional roles were included based on their universal coverage by healthcare payers in Argentina.

Nursing visits are essential for achieving care objectives, including symptom management, preventing hospital admissions and enabling patients to die at home under appropriate care [10, 13, 14]. Additionally, the well-documented shortage of nursing staff necessitates the development of strategies to optimise this valuable human resource [15, 16]. In Argentina, nurses are not authorised to prescribe medications nor therapeutic or diagnostic medical procedures; therefore, patients with high nursing care needs must also receive medical visits [17].

According to recent local data [18], there is a shortage of at least 100,000 nurses required to ensure adequate healthcare coverage. Argentina currently has an average of fewer than five nurses per physician, when the ideal ratio would be at least double that figure. This imbalance places a significant burden on the existing nursing staff and makes it more difficult to sustain the quality of care.

According to the latest data from the Federal Health Human Resources Survey (2021) [18] by the Argentine Ministry of Health:

- There are approximately 1.6 nurses per physician in the national health system (including registered nurses, technicians and nursing assistants).
- When considering only licensed registered nurses, the ratio drops to 0.5 per physician.

In contrast, the Pan American Health Organisation and the World Health Organisation recommend a minimum ratio of two nurses per physician and many countries aim for ratios between 3 and 5 to ensure adequate and sustainable care [19].

In this context, the present study aims to support a more efficient use of existing resources by guiding the appropriate allocation of nursing visits according to patient needs.

To determine the appropriate number of physician and nursing visits required for a patient, three key parameters were integrated, reflecting the multidimensional needs of patients receiving HPC. The selected parameters are based on readily available data from patient medical records:

1. **Nursing practices: NS: **This score was developed de novo based on 15 years of experience working in home-based palliative care. It includes categories of care needs that, in daily practice, require nursing interventions. These categories were scored according to their complexity and their estimated impact on the required number of nursing visits. Both clinical and social/family factors were considered, such as caregiver preparedness and the need for training in medication administration. Practices provided on a sporadic basis (e.g., enemas, bisphosphonate administration) were excluded.
2. **Symptom burden: distress score based on ESAS: **The ESAS, routinely used by the palliative teams, measures distress through 9–11 symptoms. Assuming that a higher total symptom severity score indicates a greater need for physician and nursing visits, this approach aims to enhance symptom control, reduce hospitalisations and minimise emergency department visits [20, 21].
3. **Functionality and prognosis; ECOG scale: **Performance status at the initiation of HPC has been identified as a prognostic factor [22]. The medical record routinely includes the ECOG scale, which assesses the functional status of cancer patients. This scale is particularly valuable for predicting overall survival and other clinical outcomes across various cancer types [23, 24].

The PCPT was tested by the team both at patient admission and during follow-up when changes in condition occurred. Given its satisfactory performance, a validation process was initiated.

In this first phase, the objective was to validate the **NS,** as it is a scale specifically designed by our team.

## Materials and methods

### Expert panel

The experts participating in the Delphi study (Table 1) were selected among representatives of organisations related to home palliative care. Selection criteria included:

- Broad expertise in clinical and management domains.
- Active involvement as key stakeholders in the regional development of palliative care.
- Postcollege training in palliative care (master's degree or a clinical specialty).
- At least 5 years of experience in home-based palliative care.

### Preparation: development of the NS

In the first unstructured round, a team of experts discussed which items to include in the score, based on factors identified through interdisciplinary discussions among professionals from multiple cities. The objective was to define elements that influence performance in home palliative care, particularly regarding outcomes such as place of death and frequency of hospital readmissions.

The NS includes patient-related factors that, based on the clinical experience of the home palliative care team, influence the nurse’s health care burden—whether in terms of time per visit or the total number of visits per day—required to achieve the goals of care. If nursing care is not adjusted to the patient’s needs, it may lead to hospital admission and/or hinder the possibility of dying at home. The percentage of home-based deaths and the number of avoidable hospitalisations, are quality indicators used to assess team performance [25–27].

To develop the **NS,** these factors were divided into seven categories. Each category includes multiple specific **problems** (Table 2):

1. **Parenteral access** defined using any parenteral route (subcutaneous, intravenous, and intramuscular) to administer medication and/or hydration. The indication of palliative sedation therapy, given its nature, is included as a need for parenteral access.
2. **Tubes** defined by the presence of urinary catheter, gastrostomy and/or nasogastric tubes. These three catheters were included, given that they are the most common for our team. But the category does not exclude other types of tubes, like pleural or postsurgical drainage.
3. **Simple wound care** defined by the presence of pressure ulcers grade I (intact skin with non-blanchable redness) or grade II (involves partial-thickness skin loss where the dermis is exposed).
4. **Complex wound care** defined by the presence of pressure ulcers grade III (involves full-thickness skin loss, extending to the subcutaneous tissue) or grade IV (full-thickness skin loss with exposed bone, tendon or muscle).
5. **Hygiene:** includes hygiene and comfort measures for patients that are in general (but not exclusively) bedridden. Involves, for example, regular bathing or sponge baths, maintaining oral hygiene, managing incontinence and changing bedding and clothing frequently. It also includes ensuring proper hair and nail care, changing diapers, keeping the skin clean and dry and providing a comfortable and hygienic environment.
6. **Delirium:** formerly known as acute confusional syndrome, is defined as a sudden, temporary and usually reversible disturbance in attention, cognition and consciousness level.
7. **Social/family issues:** home care depends largely on family collaboration. Therefore, problems inherent to family dynamics or arising from the patient's care, which place a burden on the family, can disrupt the continuity of care at home. The problems included by our team were discharge after prolonged hospitalisation (≥15 days), initiation of high-risk medication, patient or primary caregiver psychiatric disorder and ineffective (unprepared) family caregivers. In these situations, a more intensive nursing intervention providing support and training for caring could avoid readmissions and family distress.

If a patient presents with any of these problems, the corresponding Category score is added to their total. The **maximum possible score is 16 points,** with a **minimum score of 0. As it is shown in** Table 2.

### First and second Delphi rounds

**Figure 1.**
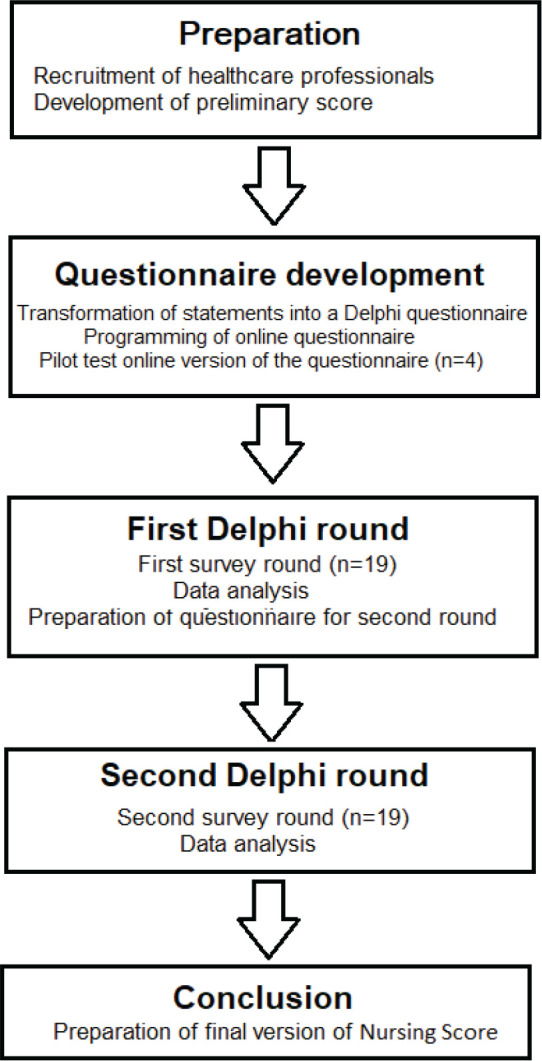
Flowchart of the Delphi process used for the development and validation of the Nursing Score.

Before initiating the Delphi rounds, the categories of the NS were operationalised into a Delphi-style questionnaire (Figure 1). The response format was a 10-point Likert scale (1 = strongly disagree, 10 = strongly agree). A pilot test of this initial version of the form was conducted with four experts to assess the clarity of the items and the feasibility of analysing the responses. Feedback from this pilot phase was used to refine the wording of the statements and to ensure the questionnaire’s usability and alignment with the objectives of the consensus process.

### Procedure

Experts were asked via Google Forms to rate each item on the Likert scale, reflecting their level of agreement. Experts received the questionnaire via email and a reminder once a week for a total period of 1 month for each round. All responses were anonymous to ensure the independence of opinions and to avoid bias due to participants' professional reputation.

### Definition of consensus and data analysis

A priori, consensus was defined as at least 80% of participants rating the item with a score of 7 or higher. Categories that failed to reach consensus after four rounds and showed no significant variation across rounds were considered for exclusion from the score.

We finally completed two rounds; the second round included the categories with the lowest degree of inter-rater agreement. As is common practice in Delphi studies, we summarised responses using medians and interquartile ranges (IQRs) to describe the central tendency and dispersion of agreement across rounds. This approach is recommended for ordinal data and provides a robust, non-parametric summary of expert consensus. The medians represent the experts’ level of agreement with each proposed item or statement. The IQR is used to indicate the degree of consensus—narrow IQRs suggest high agreement among experts. Measures of concordance or reliability are important, but these are typically applied when evaluating inter-rater reliability or internal consistency of responses. In the present study, the primary goal of the Delphi process was content validation and expert consensus, for which medians and IQRs are appropriate and commonly used. After each round, aggregated results were shared with the panel, including the median and IQR for each item, as well as the percentage of agreement (scale rate ≥ 7). Items that reached consensus in a given round were not included in subsequent rounds.

Statistical analysis was performed using R software version 4.3.0. In addition to the Delphi analysis, Aiken’s V coefficient was calculated to assess the degree of agreement among experts regarding the relevance of each item for inclusion in the score. This statistical index quantifies the content validity based on expert ratings and was used to support the evaluation of each item's appropriateness alongside the consensus criteria. A *p*-value < 0.05 was considered significant.

## Results

During the study to reach consensus on the **NS**, two Delphi rounds were conducted with 19 expert palliative care professionals from Argentina. The experts were selected based on their experience in home-based palliative care and postgraduate academic training. The panel consisted of 13 physicians and six nurses who met the specified criteria, representing nine cities across six different provinces (Table 1).

The results of the content validation analysis using the Aiken V test indicate high levels of concordance between experts for all the categories evaluated. The categories ‘Simple Wound care’, ‘Complex Wound care’ and ‘Socio-family Issues’ obtained a perfect Aiken V index (1.00) with statistical significance (*p* < 0.001), reflecting a solid validation of these dimensions. On the other hand, the categories ‘Parenteral access,’ ‘Tubes,’ ‘Hygiene’ and ‘Delirium’ presented values of 0.95, also with statistical significance (*p* < 0.001). The results of the Aiken methodology (Aiken V test) are shown in Table 3.

These results suggest that the proposed instrument has high content validity, supporting its use in clinical practice.

The analysis of the two Delphi rounds, carried out using R software version 4.3.0, achieved a high degree of consensus among the experts. In the first round, the median scores ranged between 7 (P25: 5, P75: 10) and 10 (P25: 8.5, P75: 10). In the second round, adjustments in the median and greater homogeneity in the responses were observed, highlighting an increase in agreement with values higher than 84% for all categories. Two items reached 100% agreement and four others exceeded 90% consensus. These results show that the Delphi method was effective in refining and validating the dimensions of the NS, guaranteeing robust consensus among the participants (Table 4).

## Discussion

The PCPT was specifically designed for the home palliative care setting in Argentina, with the aim of assigning the number of professional visits based on an objective criterion. Since the NS was developed ad hoc for this tool, we consider its validation essential as a first step.

Community palliative care models that include regular home visits show a positive impact on patient outcomes and quality of life [10]. These models emphasise the importance of frequent and well-planned visits to address symptom distress and improve daily functioning, particularly in patients in rehabilitation [28].

The literature suggests a flexible approach to visits, with adjustments based on patient needs and goals of care. In general, the frequency of home palliative care visits is determined by a combination of patient-specific factors, such as disease progression and symptom burden, and health system-level factors, such as resource availability and cost considerations. Since evidence shows that decision-making regarding the number of home visits is often subjective [15, 29], the use of standardised tools may be of great use [11]. These tools improve the efficient allocation of resources while maintaining high quality of care [30, 31].

The study by Unroe *et al* [32] highlights that the intensity of nursing care tends to be higher at the beginning and end of palliative care follow-up, which may reflect the need for more intensive management during these critical periods. This pattern suggests that health care professionals may adjust the frequency of visits based on the patient's stage of illness and anticipated need for symptom management and support [32].

Seow *et al* [14] found that an increase in home nursing care, particularly with a focus on the end of life, was associated with a reduction in hospitalisation rates, indicating that more frequent nursing visits may be beneficial in preventing hospital admissions during the last months of life [14]. This finding supports the idea that visit frequency can be tailored to reduce acute care utilisation and support patients to remain at home.

As limitations of the present study, we should note several important concerns regarding its applicability and effectiveness in real-world settings. First, the small sample size—19 professionals from a single country, most of whom are affiliated with the same home care team—may limit the generalisability of the findings. Additionally, the Delphi panel included a larger number of physicians (13) than nurses (6), despite the tool being an NS. The observed imbalance primarily stemmed from the availability of a dedicated research group. This group, although mainly composed of physicians, consisted of individuals who all met the study's rigorous selection criteria: extensive experience in home-based palliative care and postgraduate academic training in palliative care. While we acknowledge this as a limitation, the collective expertise and qualifications of all panel members were paramount in ensuring the rigor and validity of the score's development. Furthermore, it is important to incorporate feedback from patients and caregivers into the development and validation process to ensure that the tool aligns with the real needs and preferences of those receiving care. Finally, in complex and nuanced fields such as palliative care, human judgment and individualised care plans should remain a priority, always considering each patient’s unique circumstances. Future research should explore this tool and incorporate a broader range of stakeholder perspectives.

## Conclusion

The NS is a valid instrument that, when integrated with other scales, allows for an objective determination of the number of visits by nurses and physicians in home palliative care for the adult oncology population in Argentina.

Decision-making on the number of home visits, based on parameterisation tools such as PCPT, aligns with what is suggested by the literature and offers a robust framework for optimising the provision of care. Future studies are needed to validate the rest of the components of the tool.

## Conflicts of interest

The authors declare no conflicts of interest.

## Funding

This research did not receive any specific grant from funding agencies in the public, commercial or non-profit sectors. The study was carried out as part of the authors’ routine professional activities within CAREHOME.

## Author contributions

Agustín Flores, Alejandra San Martín, Carina Badalotti, Carolina Ghiglieri, Constanza Nuñez, Diego Scotta, Gabriela Capalbo, Gabriela Nuñez, Graciela Jury, Jacqueline Cimerman, Lorena Aranda, Lucas Bode, María del Carmen Fernandez, Mariangeles Fossatti, Martín Nallar, Melina San Segundo, Mónica Buriano, Sandra Vela Pérez, Silvia Lazarte.

## Ethical considerations

This study did not involve patients or personal data. All participants were healthcare professionals voluntarily completing an anonymous survey. Therefore, ethical approval was not required, in accordance with national guidelines.

## Declaration of generative AI & AI-assisted technologies in the writing process

Artificial intelligence (AI) tools, specifically ChatGPT (OpenAI, GPT-4), were used in the preparation of this manuscript to improve the clarity of English grammar, spelling and language structure. These tools were employed to enhance the readability and presentation of the text but were not used to generate scientific content, interpret data or influence the study's conclusions.

## Figures and Tables

**Table 1.** Characteristics of the surveyed experts.

*Category*	*Quantity*	*Percentage*
*Professionals surveyed*		
*Physicians*	13	68.4%
*Nurses*	6	31.6%
*Background in palliative care*		
*Specialist in palliative care*	8	42.1%
*Master in palliative care*	1	5.3%
*Postgraduate in palliative care*	10	52.6%
*Years of experience in home palliative care*		
*5–10 years*	1	5.1%
*>10 years*	18	94.9%

**Table 2.** Nursing score of the PCPT.

*Nursing score*			
*Category*	Problem	Score	Total
*Parenteral access*	Subcutaneous for hydration	5	5
	Parenteral medication (EV, IM, SC)		
	Palliative sedation therapy		
*Tubes*	Urinary catheter	1	1
	Nasogastric tube		
	Gastrostomy		
*Simple wound care*	Grade I pressure ulcers	1	1
	Grade II pressure ulcers		
*Complex wound care*	Enterocutaneous fistula	2.5	2.5
	Other fistulas		
	Tracheostomy		
	Colostomy		
	Grade III pressure ulcers		
	Grade IV pressure ulcers		
*Hygiene*	Hygiene and comfort	2	2
*Delirium*	Delirium	2.5	2.5
*Social/family issues*	Initiation of opioid therapy (training required)	2	2
	Initiation of high-risk medication (anticoagulation, insulin, others)		
	Discharge after prolonged hospitalization (≥15 days)		
	Ineffective family caregivers		
	Patient with psychiatric disorder		
	Primary caregiver with psychiatric disorder		
	Total		16

**Table 3.** Validation of the nursing score content by Aiken's V test.

	*V Aiken*	*p-value*
*Parenteral access*	0.95	<0.001
*Tubes*	0.95	<0.001
*Simple wound care*	1.00	<0.001
*Complex wound care*	1.00	<0.001
*Hygiene*	0.95	<0.001
*Delirium*	0.95	<0.001
*Social/family issues*	1.00	<0.001

**Table 4.** Analysis of the 2 Delphi rounds with R software version 4.3.0.

	Round 1	Round 2	Final
*Category*	Median (IQR1–IQR3)	Median (IQR1–IQR3)	% Agreement ≥ 7
*Parenteral access*	10 (8.5–10)		16 (84.2%)
*Tubes*	7 (5–10)	7 (7–8.5)	16 (84.2%)
*Simple wound care*	8 (7–10)		16 (84.2%)
*Complex wound care*	9 (8–10)		17 (89.5%)
*Hygiene*	8 (5–10)	8 (8–8)	100%
*Delirium*	10 (6.5–10)	10 (10–10)	18 (94.7%)
*Social/family issues*	10 (7.5–10)		16 (84.2%)

## References

[1] Radbruch L, De Lima L, Knaul F (2020). Redefining palliative care—a new consensus-based definition. J Pain Symptom Manage.

[2] Chung HH, Wang CL, Wu JJ (2024). Trend analysis of quality indicators in palliative home care among terminally ill cancer and non-cancer patients in Taiwan: a 6-year observational study. Supportive Care Cancer.

[3] Maetens A, Deliens L, Van den Block L (2019). Are we evolving toward greater and earlier use of palliative home care support? a trend analysis using population-level data from 2010 to 2015. J Pain Symptom Manage.

[4] Sun Z, Laporte A, Guerriere DN (2005). Utilisation of home-based physician, nurse and personal support worker services within a palliative care programme in Ontario, Canada: trends over 2005–2015. Health Soc Care Community.

[5] Cherro AF (2024). Cost-saving with home palliative care: the trend in some developed nations and the current situation in Argentina. Nursing Commun.

[6] Luta X, Ottino B, Hall P (2021). Evidence on the economic value of end-of-life and palliative care interventions: a narrative review of reviews. BMC Palliat Care.

[7] Irani E, Hirschman KB, Cacchione PZ (2018). Home health nurse decision-making regarding visit intensity planning for newly admitted patients: a qualitative descriptive study. Home Health Care ServQuart.

[8] Jack BA, O'Brien MR, Scrutton J (2015). Supporting family carers providing end-of-life home care: a qualitative study on the impact of a hospice at home service. J Clin Nurs.

[9] Sockolow PS, Bowles KH, Pankok C (2021). Planning the episode: home care admission nurse decision-making regarding the patient visit pattern. Home Health Care Manage & Pract.

[10] Allo JA, Cuello D, Zhang Y (2016). Patient home visits: measuring outcomes of a community model for palliative care education. J Palliat Med.

[11] Demirbilek M, Branke J, Strauss A (2019). Dynamically accepting and scheduling patients for home healthcare. Health Care Manag Sci.

[12] Harrold J, Byhoff E, Harris P (2014). All hospice patients are not equal: development of a visit-based acuity index. J Palliat Med.

[13] Seow H, Sutradhar R, Mcgrail K (2016). End-of-Life Cancer Care: temporal Association between Homecare Nursing and Hospitalizations. J Palliat Med.

[14] Sutradhar R, Barbera L, Seow HY (2017). Palliative homecare is associated with reduced high- and low-acuity emergency department visits at the end of life: a population-based cohort study of cancer decedents. Palliat Med.

[15] Boniol M, Kunjumen T, Nair TS (2022). The global health workforce stock and distribution in 2020 and 2030: a threat to equity and ‘universal’ health coverage?. BMJ Glob Health.

[16] Pastrana T, Torres-Vigil I, De Lima L (2014). Palliative care development in Latin America: an analysis using macro indicators. Palliat Med.

[18] https://www.boletinoficial.gob.ar/detalleAviso/primera/293212/20230831.

[19] (2023).

[20] Stapleton SJ, Holden J, Epstein J (2016). A systematic review of the symptom distress scale in advanced cancer studies. Cancer Nurs.

[21] Hannon B, Dyck M, Pope A (2015). Modified edmonton symptom assessment system including constipation and sleep: validation in outpatients with cancer. J Pain Symptom Manage.

[22] Mercadante S, Valle A, Porzio G (2013). Prognostic factors of survival in patients with advanced cancer admitted to home care. J Pain Symptom Manage.

[23] Kumar D, Neeman E, Zhu S (2024). Revisiting the association of ECOG performance status with clinical outcomes in diverse patients with cancer. JNCCN J Nat Comprehensive Cancer Netw.

[24] Käsmann L, Taugner J, Eze C (2019). Performance status and its changes predict outcome for patients with inoperable stage III NSCLC undergoing multimodal treatment. Anticancer Res.

[25] Chang PJ, Lin CF, Juang YH (2023). Death place and palliative outcome indicators in patients under palliative home care service: an observational study. BMC Palliat Care.

[26] Busquet-Duran X, Jiménez-Zafra EM, Manresa-Domínguez JM (2020). Describing complexity in palliative home care through hexcom: a cross-sectional, multicenter study. J Multidiscip Healthc.

[27] Corrêa MI, Bruera E, Alves JDO (2025). Transforming end-of-life care: impact of the “Melhor em Casa” home-based palliative care program in Brazil. J Palliat Med.

[28] Shimoda K, Imai H, Tsuji T (2019). Factors affecting the performance of activities of daily living in patients with advanced cancer undergoing inpatient rehabilitation: results from a retrospective observational study. J Phys Ther Sci.

[29] Pastrana T, De Lima L (2022). Palliative care in Latin America: are we making any progress? assessing development over time using macro indicators. J Pain Symptom Manage.

[30] Gallastegui-Brana A, Rodríguez-Nunez A, Palacios J (2023). Development and validation of a tool to assess the structural quality of palliative care services. J Pain Symptom Manage.

[31] Boddaert MS, Douma J, Dijxhoorn AFQ (2022). Development of a national quality framework for palliative care in a mixed generalist and specialist care model: a whole-sector approach and a modified Delphi technique. PLoS One.

[32] Unroe KT, Bernard B, Stump TE (2017). Variation in hospice services by location of care: nursing home versus assisted living facility versus home. J Am Geriatr Soc.

